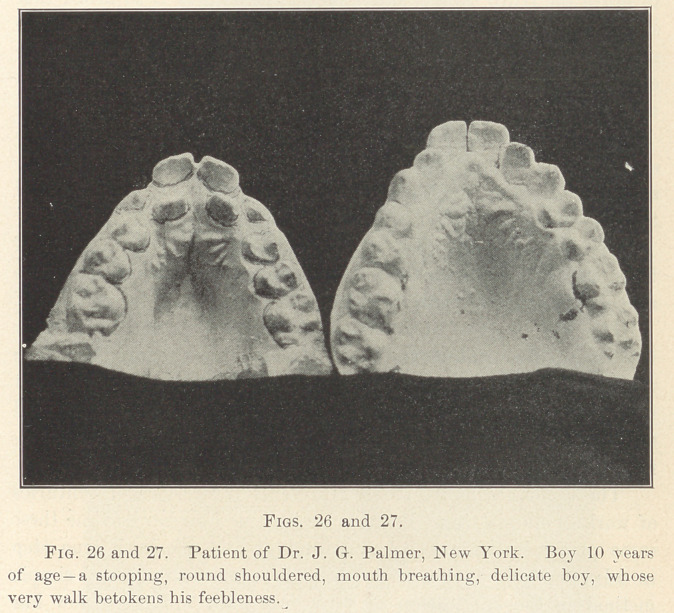# The Influence, on Development, of Arranging Irregularly Placed Teeth into Normal Positions1Read at the annual meeting of the British Medical Association at Leicester, July 28, 1905.Republished from the Journal of the British Medical Association, with added illustrations.

**Published:** 1905-12

**Authors:** E. A. Bogue

**Affiliations:** New York City


					﻿THE
International Dental Journal.
Vol. XXVI.
December, 1905.
No. 12.
Original Communications.
THE INFLUENCE, ON DEVELOPMENT, OF ARRANGING
IRREGULARLY PLACED TEETH- INTO NORMAL
POSITIONS.1
1 Read at the annual meeting of the British Medical Association at Leicester,
July 28, 1905.
Republished from the Journal of the British Medical Association, with added
illustrations.
BY E. A. BOGUE, M.D., D.D.S., NEW YORK CITY.
Before starting to consider the question that I am about to
bring before you, let me say that I have not been unmindful of the
influence of adenoid vegetations in producing the irregularly placed
teeth which are so generally, if not uniformly, found in connection •
with mouth breathing, high arches and nasal stenosis, but all that
has been purposely left out, that our attention may be drawn to
certain features that have been less discussed, if indeed discussed at
all.
I am clearly of the opinion that neglect is at the bottom of the
worst cases we have to treat, that early treatment of the conditions
now to be considered will result in the prevention of much of the
evils of those conditions, and that preventive medicine is the highest
level of that art.
Mr. President and Gentlemen : I was invited by your com-
mittee to furnish something of dento-surgical interest to be read
at this meeting, for instance, “ The Influence of Development on
the Arrangement of the Teeth.” This invitation furnishes me with
the title of my subject, which will be “ The Influence, on Develop-
ment, of Arranging Irregularly Placed Teeth into Normal Posi-
tions.”
Many of the evils that we as orthodontists are called upon to
treat, and many of the diseases which confront the rhinologist,
arise from a deficiency in the vital force of the child—an inability
to overcome some mechanical obstruction.
Every developmental process is an expression of vitality. Every
defect in formation or function is an indication of a deficiency in
a motor centre of power sufficient to overcome obstructions. The
expression of deficiency is various.
One of the most common expressions, and one that every dental
surgeon and rhinologist should recognize, is irregularity in the
arrangement of the teeth of the child. Sometimes this irregularity
shows itself in the temporary teeth, sometimes it shows first in the
malarticulation of the first permanent or principal molars.
The lower principal molars of man have upon their outer or
buccal side three cusps or tuberosities; upon the inner or lingual
side they have two.
The upper molars have two on either side. In the normal
position of these teeth when closed tlie anterior buccal cusp of the
upper molar and the anterior lingual cusp sit astride of the outer
ridge of the lower first molar, just posterior to, and nicely fitting in
with, the anterior buccal cusp of the lower molar.
This arrangement is made necessary by the greater width of the
upper incisor teeth over the lower incisors.
The same general arrangement exists in the case of the tempo-
rary teeth, so that the careful observer may detect irregularities in
the positions ot the temporary teeth that are sure indications of
malpositions among the permanent teeth that are not yet erupted.
Whenever there has been loss of temporary teeth, or even exces-
sive decay, and a loss of considerable portions of the temporary
teeth, the principal molars, which are either in the process of
development or are just erupting, are pressed forward by the erup-
tive processes and seek to occupy the places of tissue lost by decay
or of the missing temporary teeth.
For example, a temporary tooth becomes decayed, the pulp is
exposed and dies. The process of absorption of the roots, so far as
such absorption is dependent upon the pulp, ceases, solution from
whatever cause is slower, hence this pulpless tooth is an obstacle to
the regular development of the tooth beneath, and if this tooth suc-
ceeds in emerging from beneath the temporary tooth it is deflected
from its proper place in the arch.
If the cavity of decay be upon the posterior approximal side of
the temporary tooth, toward the erupting first molar, this first
molar sometimes leans forward into such a cavity enough to lose
its proper position and its antagonism with the lower molar.
If, as in the illustration, the last temporary molar is lost, and
the first molar is also decayed on its posterior approximal surface,
the tendency of the incoming first permanent molar is to occupy
both vacancies and to come forward the full width of a tooth or
more.
Not only will it come forward, but in coming forward it ad-
vances on the lines of the arch of the jaw, and the further forward
it comes the narrower becomes the space between it and its opposite
molar on the same jaw.
These principal molars occupy m the adult normal jaw the
middle of the arch from before backward between the cuspid and
the wisdom tooth.
As they are in a sense the keystones of these antero-posterior
arches they become, upon their eruption, the keys to the situation,
for if these four keystones or pillars to the dental arches are
normally arranged at the period of their development they support
the jaws during the shedding of the temporary teeth and their re-
placement by the permanent ones, and we are almost certain that
all the other molars posterior to these will be correctly arranged.
It is also almost certain that the teeth anterior to these keystones
with whose development and proper placing in their alveoli we have
to deal may be easily inducted into their places, should they erupt
irregularly, practically without pain, and therefore without detri-
ment to the well being of the child, whose growth is not thereby
inhibited, and whose usual avocations are but slightly interfered
with.
These permanent molars, then (let us suppose a case of upper
teeth), which have been deflected from their normal positions,
occupy a certain amount of room (space) anterior to what they
should, and consequently they crowd out of place the other perma-
nent teeth anterior to the molars, either by a narrowing of the arch
and a protrusion of the incisors, or a bunching of the bicuspids
or both.
For it must be remembered that the teeth of any given individ-
ual are adapted to the size of that individual’s jaws and alveolus.
(There is no such thing as large teeth in small jaws and alveoli,
for the alveolus is formed upon the jaws after the crowns of the
teeth are complete, and it forms around the roots of those teeth
that are themselves in process of formation.)
But there is still further mischief caused by the decay of these
temporary teeth.
The development or displacement of even one permanent molar
further forward on the arch than it should be, therefore narrowing
the arch, confines and cramps to a considerable extent all those con-
tiguous bones that are in process of development at just this period,
and obstructs their growth; so that unless aid is rendered and the
mechanical obstruction is removed, the face never gets its full
development, the vault of the palate is more or less deformed,
there is not room for the tongue, still less for the free use of it,
nor for vocal resonance, which fails unless there is ample room
and a broad and well-formed palatine arch; nor for full and free
inspiration, owing to a diminished nasal meatus, so that often we
find mouth breathing supervening upon the loss of certain of the
baby teeth and the development of the permanent molars too far
forward in the arch.
It will be perceived, therefore, that if at a very early age we can
positively diagnose for impending irregularities of the permanent
teeth, it will also be possible in most instances to correct those
irregularities, and without extraction, during the eruption of the
permanent teeth, beginning the operations while the temporary
teeth are still among the instruments to be acted upon, broadening
the arches and correcting the articulations of the principal molars.
If this is done at this early age not only will the growth of the
superior maxillae and the alveoli be promoted, but the growth of
the adjacent bones of the nose, palate, head and face will also be
promoted. Instead of narrow or saddle-shaped jaws with deficient
nasal passages, obstructed oftentimes by undue and excessive con-
volution of the turbinated bones and by recurring lymphoid growths,
which growths are promoted by the contracted regions in which
they lie, we shall have a resumption of growth in the maxilhe,
palate bones and nasal bones, and probably as well in the ethmoid
and sphenoid bones. The nasal septum will be straightened con-
siderably by broadening the arch of the upper jaw and so lowering
it, and as this broadening relieves the pressure and tends to
straighten the septum so proper nutrition is encouraged and Nature
is allowed to exert her influence toward a development of all the
sinuses, which become freer and more nearly normal.
As this gives better respiration, the lungs themselves become
enlarged, the strength of the patient increased, and the dignity
of expression, which comes with distinct speech, good articulation
and resonance of voice follows as a matter of course.
I will try to exhibit a few specimens of these results as I close.
I am aware of the objections raised to these views, especially on
the part of those who say that having been brought up to extract
teeth, both for children and adults for the correction of certain
irregularities, they cannot see why patients should be allowed to
seem “ all teeth” when the extraction of a few of the seemingly
superabundant ones would so easily correct the difficulty. It is evi-
dent that those who make this claim cannot have considered with
sufficient care the processes of growth and the results of a little
easily obtained expansion in the regions under discussion.
At birth the ethmoid bone is small, very incomplete, and im-
perfectly ossified. The hard palate at the height of the arch is
above the eustachian tube. In the adult the hard palate is below
this tube, consequently, as Dr. Swain, Professor of Otology and
Laryngology, Yale University, has correctly observed in one of his
papers, “ the palate has grown away from the base of the skull.
What has accomplished this? It has been the downward growth
of the superior maxillary bone, the septum and the pterygoid
processes of the sphenoid, so that in a certain sense of the word the
palate of a young child is supposed to grow down instead of up. It
is safe to assume, therefore, that this downward growth is an ex-
tremely important process in our present consideration, and it may
be looked upon as a part of the normal growth of the superior
maxillary bone.”
Now it happens that the sutures of most of the bones of the head
and face do not solidify until adult life, some of them not until
middle life; they are, therefore, during all this time subject to
change, and if expansion of irregular dental arches is accomplished
previous to solidification, it is almost always the case that, stimu-
lated by the removal of undue repression of the parts seeking de-
velopment, renewal of growth will take place, and one or two
years is quite sufficient time for the changes of such growth to
produce a nobler countenance out of a most unpromising one.
These changes can be best noticed by photographs taken before and
after the operations, by models of the teeth made at the same time
as the photographs, and if the operator so wills by plaster casts of
the face, which are easily made, and are unimpeachable witnesses
as to what occurs.
I have been working along these lines for about five years,
aiming to correct irregularities in the positions of the teeth as
early as I could get control of them, and that is often from the
fifth to the seventh year, the very climacteric in the child’s life,
when growth begins to manifest itself most strenuously. The re-
sults not only in my own hands, but in those of my son, and one of
my former pupils, Dr. Kelsey, of Baltimore, and in a few cases in
the hands of other practitioners, have gone far to convince me of
the soundness of my position not only, but of the soundness of the
hypothesis upon which I have worked.
I find by an examination of many hundreds of aboriginal skulls
in the development of which Nature has been free to work her will
without the assistance of man, and where the so-called accidents
of development occurred as they do with us civilized races, though
probably not with the same frequency, that in all those cases where
irregularities in the development and positions of the upper teeth
exist there are also irregularities, more or less strongly marked in
the development of the maxillae and the palate bones, and seemingly
in the vomer, the sphenoid and ethmoid as well, and that the nasal
septum particularly is sure to be crooked and the turbinated bones
so excessively curved as to materially interfere with the proper open-
ings of the nasal meatus. And when I say irregularities of the
upper teeth, I am not forgetting that the upper jaw with its teeth
is dependent upon the lower for correctness of position.
Per contra, by just so much as the permanent teeth are
regular in their development, and are regularly placed in their
arches, all the sinuses of the maxillae and facial bones are better
developed, the nasal septum becomes straighter, the turbinated
bones less obstructive, the sphenoid and ethmoid larger proportion-
ately to the size of the skull, and the facial bones occupy positions
that add the dignity of strength to the individual.
Illustration: John M., 8 years old; began May 6, 1900. Last
illustration made May, 1902. Fixtures remained on for two years
from the time they were placed. Actual time occupied in moving
the various irregularly placed teeth into their present positions,
sixty days interspersed in different periods during those two years.
Absolutely no pain.
Adenoids have been removed in this case prior to the en-
largement of the arch. No recurrence of that tissue. Face and
nose both show the enlargement and the teeth retain their normal
positions, breathing normal, general development rapid.
Applications of apparatus for regulating have been irregular,—
on and off almost at the will of the patient,—but the last illustra-
tion shows the stage which the boy had reached when 14 years of
age, at which time he had nearly lost his stooping shoulders, en-
tirely quit mouth breathing, strengthened and developed in a re-
markable degree, and, I am told, is now willing to wear an ap-
paratus continually. No thought of improving his breathing or
his general health was entertained when the enlargement of the
arch was undertaken.
Do not understand that the single phase that I have to-day pre-
sented is any more than one phase in conditions that have taxed
the intelligence and the patience of both rhinologists and dentists
to the last degree, but if my confreres may be helped over some few
of their perplexities by a recognition of their causes and a prompt
and early application of the simple mechanical apparatus necessary
to correct these deformities, I shall be very glad to have been the
instrument through which such a presentation has been made.
Four days after mailing this essay to your committee, I noticed
in the Dental Cosmos for May, 1905, a paper by Hofzahnartz W.
Pfaff, of Dresden, Germany, entitled “ Stenosis of the Nasal Cavity,
Caused by the Contraction of the Palate and Abnormal Position of
the Teeth; Treatment by Expansion of the Maxilla,” that had been
read at the St. Louis Congress while I was ill in the hospital, and
is along almost precisely the same lines as my paper.
I take pleasure in calling your attention to this paper as strongly
confirming the views just presented for your consideration.
As the two papers were the results of two persons, the one in
Germany, the other in America, working unknown to each other on
the same subject, the results are worthy of note.
And while both papers are far from being exhaustive, they at
least indicate a subject for further investigation.
				

## Figures and Tables

**Fig. 1 f1:**
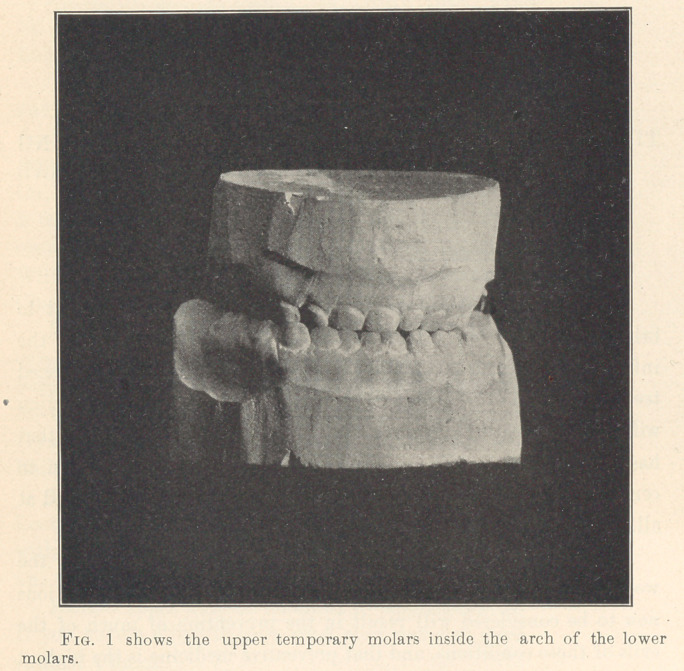


**Fig. 2 f2:**
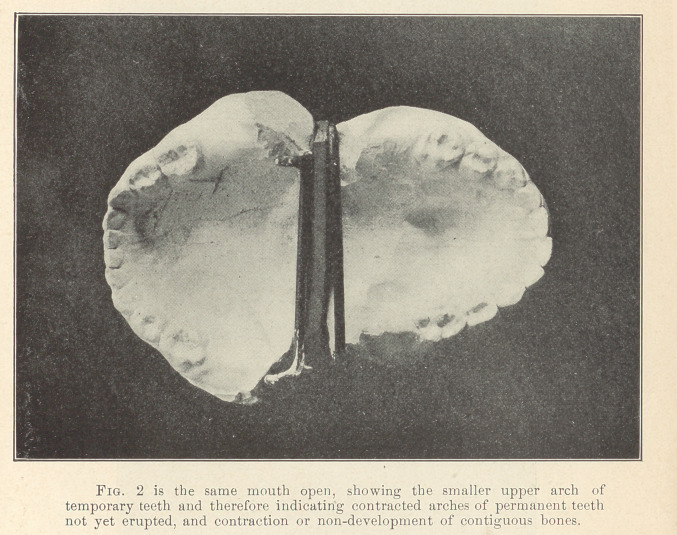


**Fig. 3 f3:**
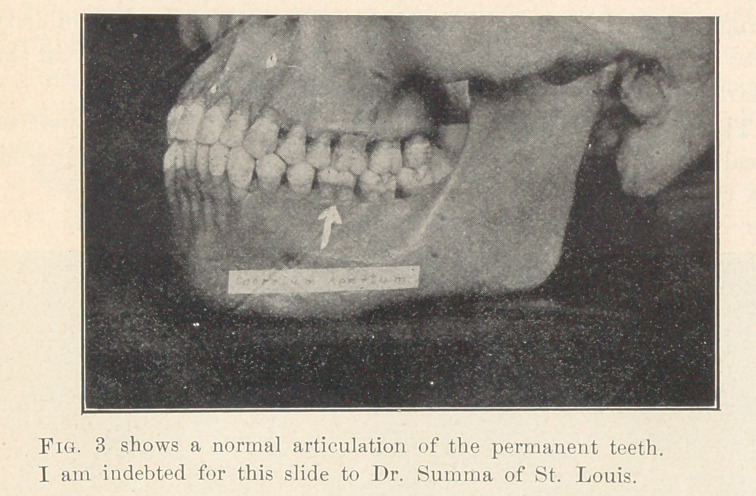


**Fig. 4 f4:**
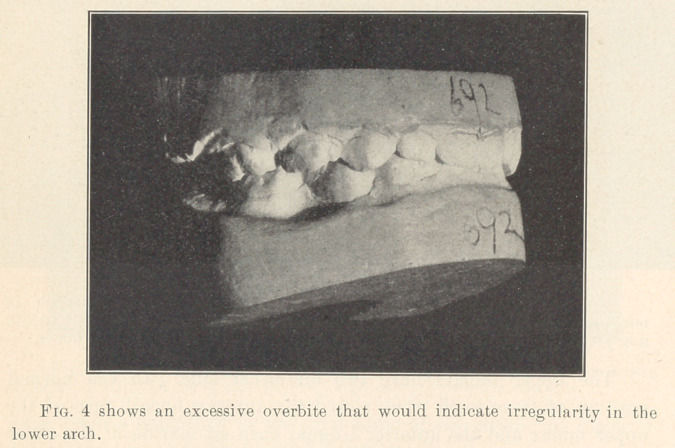


**Fig. 5 f5:**
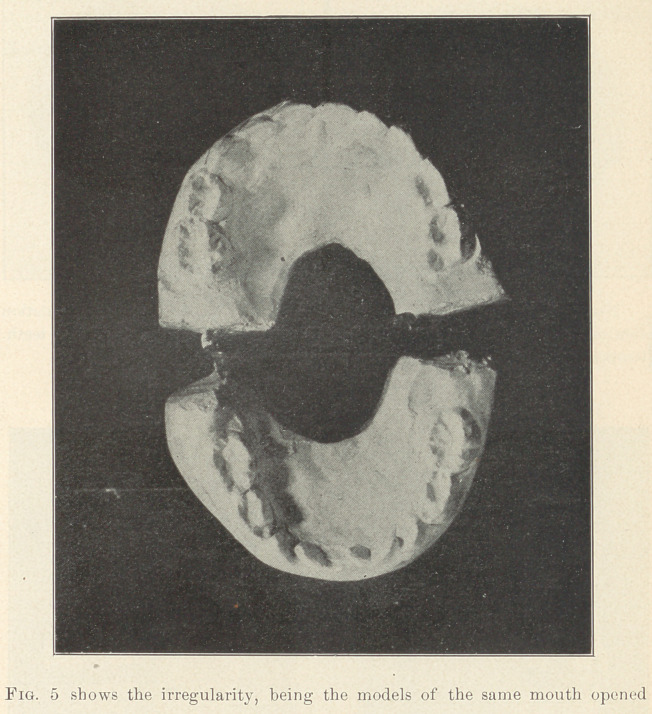


**Fig. 6 Fig. 7 f6:**
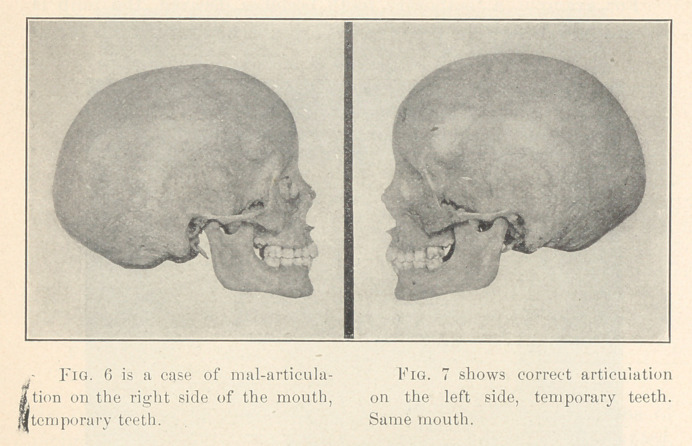


**Figs. 8 and 9 f7:**
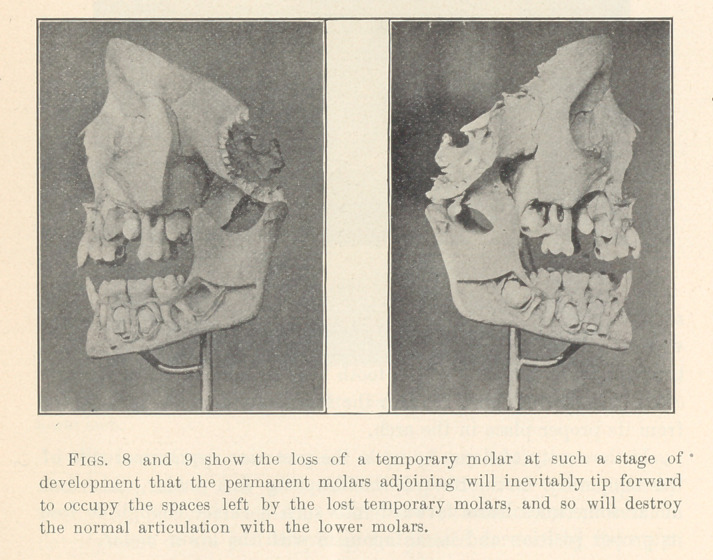


**Fig. 10. f8:**
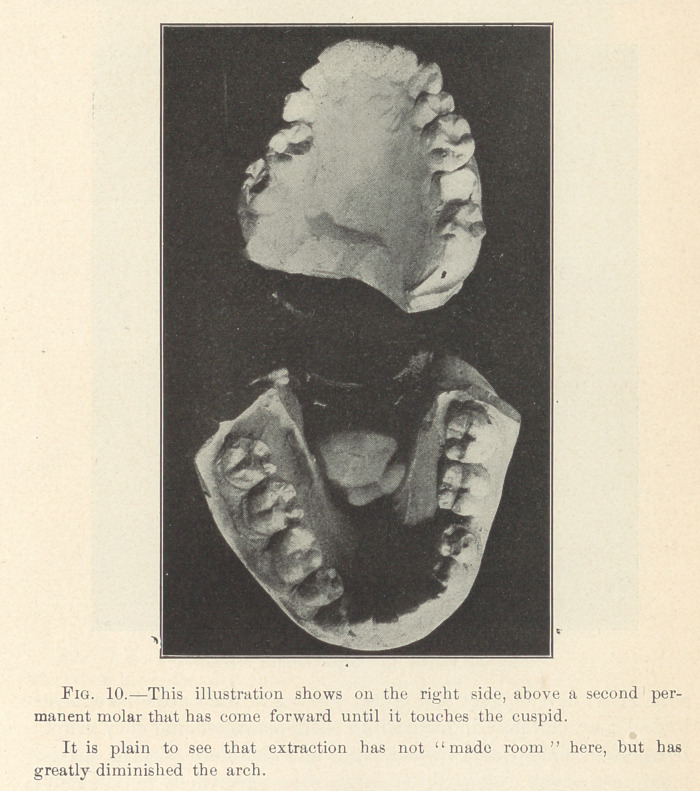


**Fig. 11 f9:**
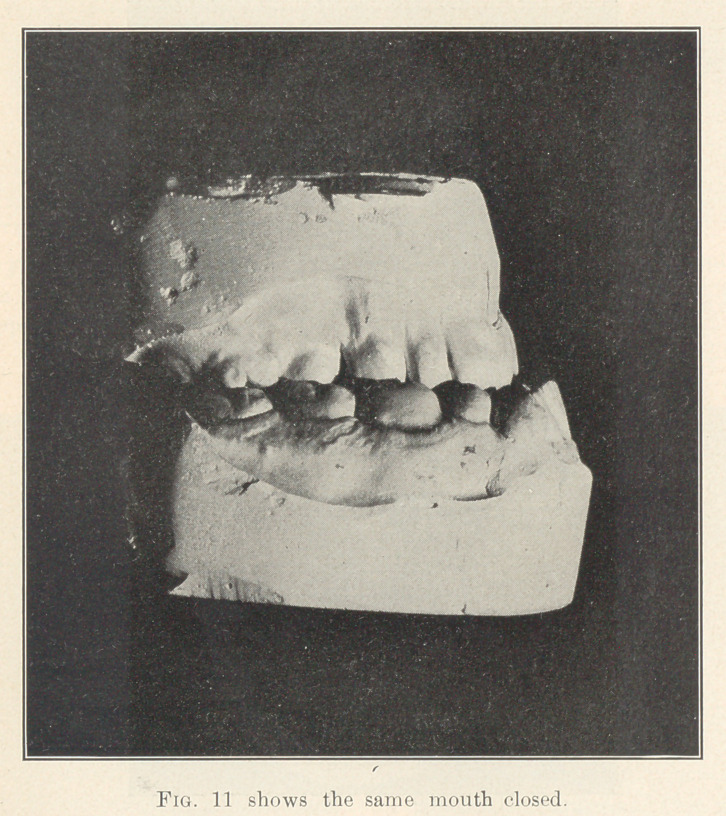


**Fig. 12 f10:**
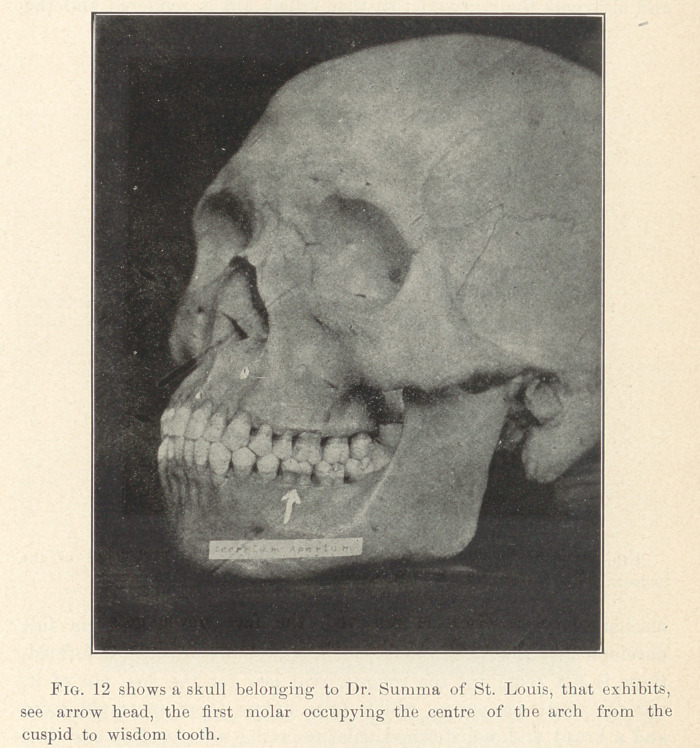


**Fig. 13 f11:**
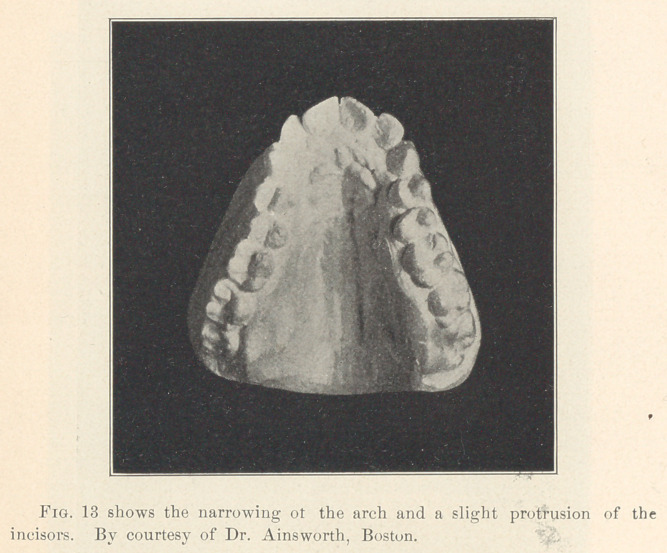


**Fig. 13 A. f12:**
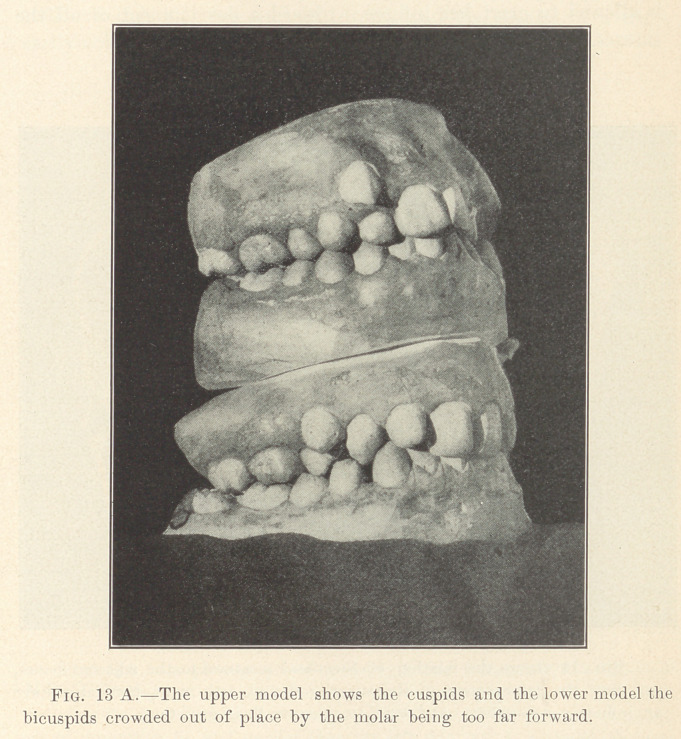


**Fig. 14 f13:**
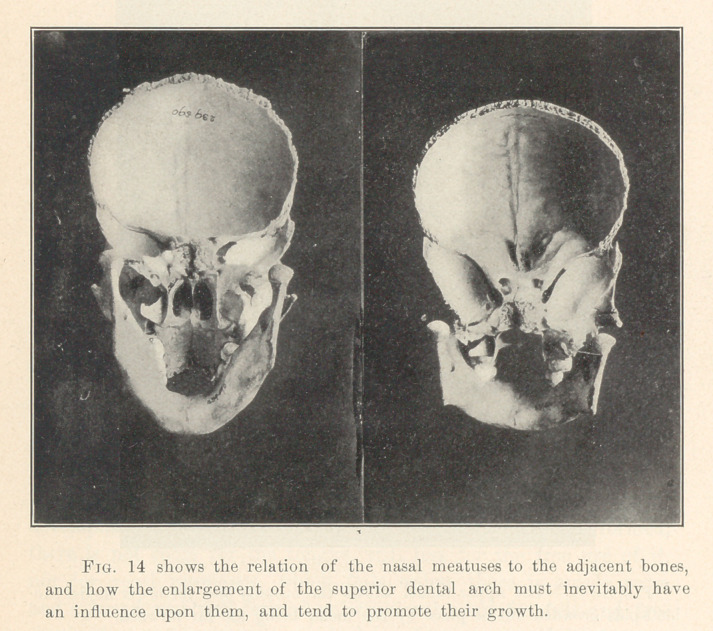


**Fig. 14A f14:**
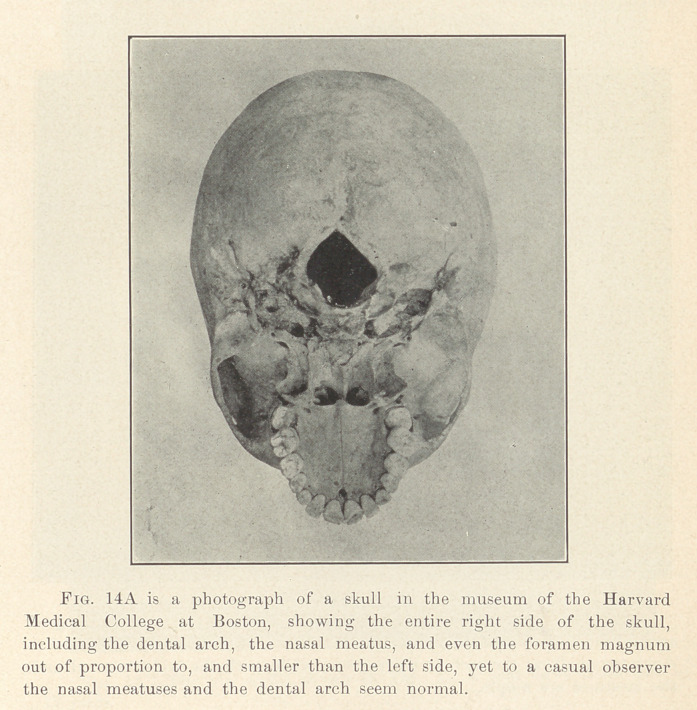


**Fig. 14B f15:**
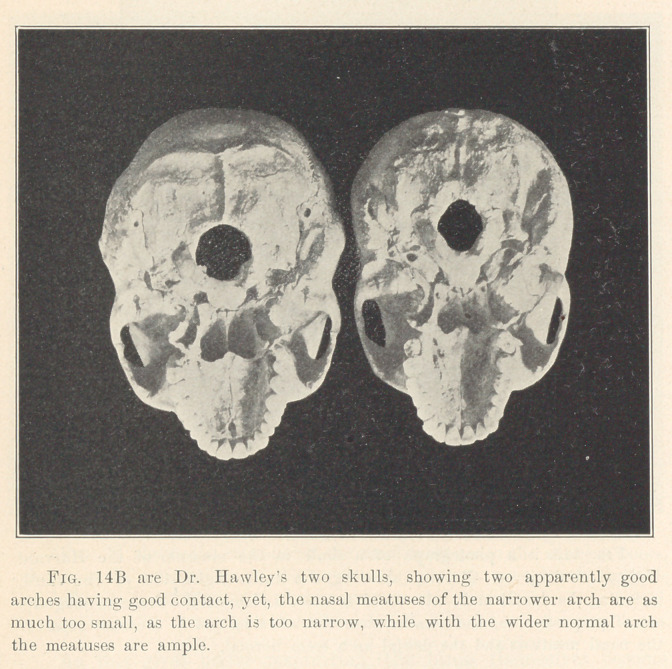


**Fig. 15. f16:**
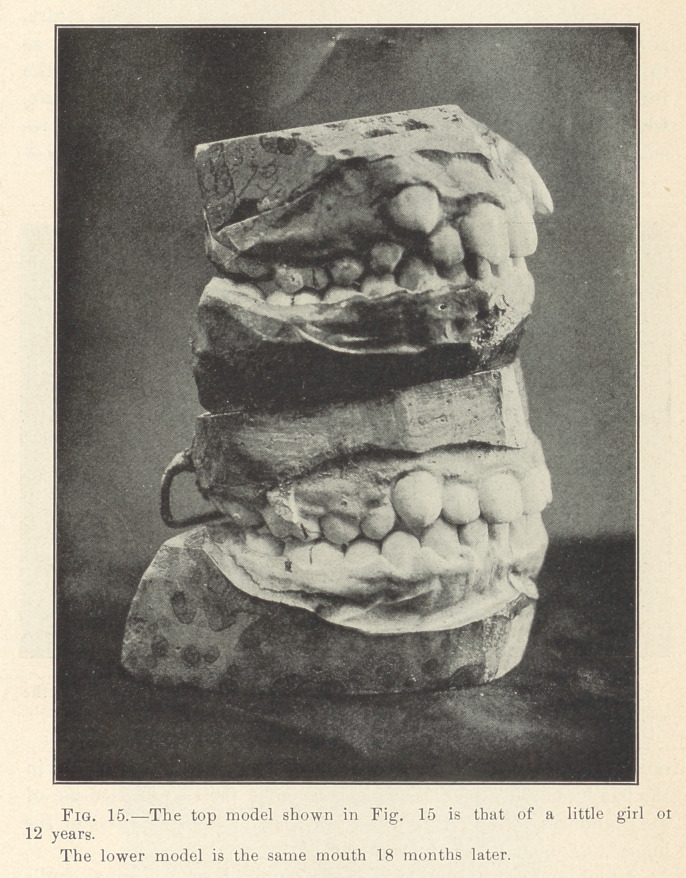


**Figs. 16 and 17. f17:**
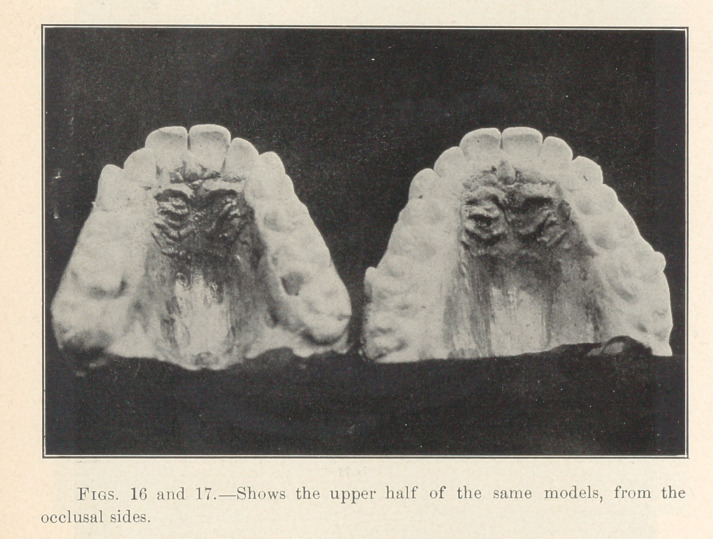


**Fig. 18. f18:**
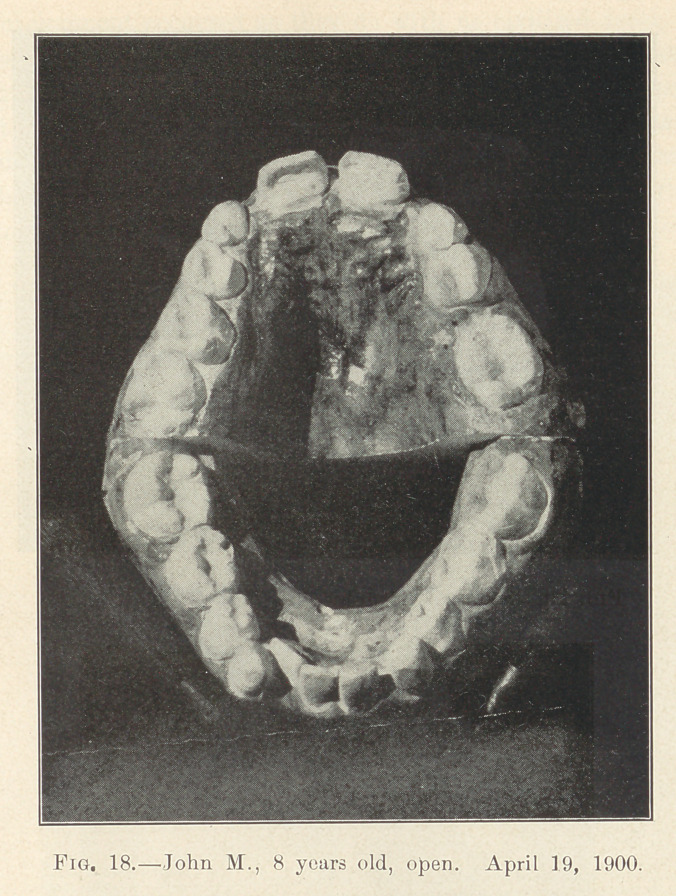


**Fig. 19. f19:**
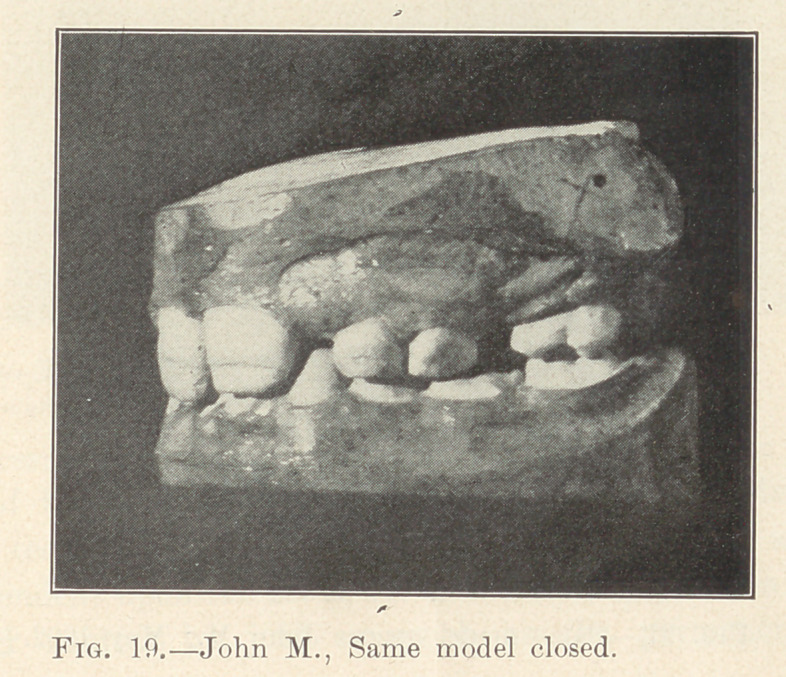


**Fig. 20. f20:**
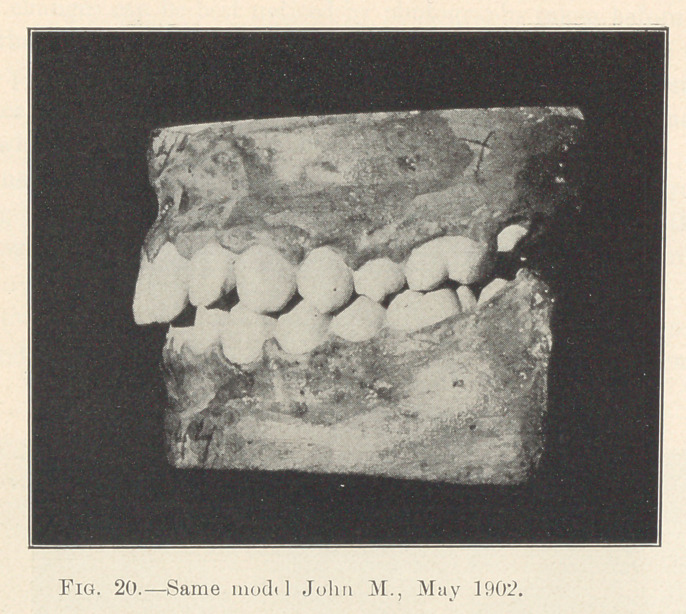


**Fig. 21. f21:**
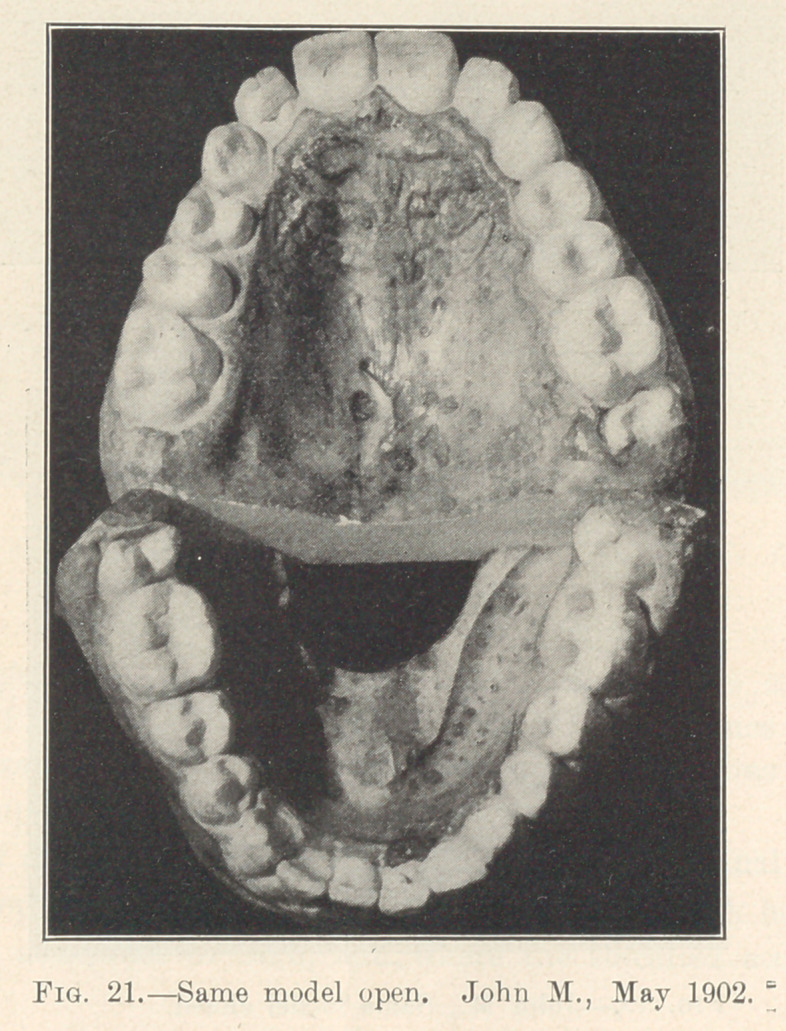


**Fig. 22. f22:**
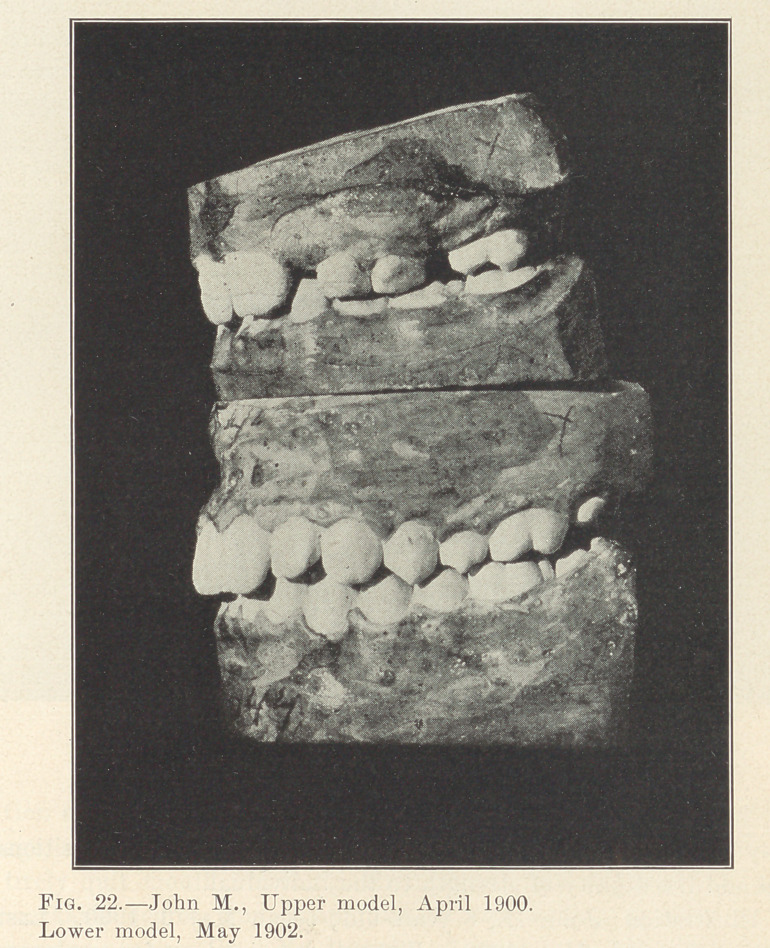


**Fig. 23. f23:**
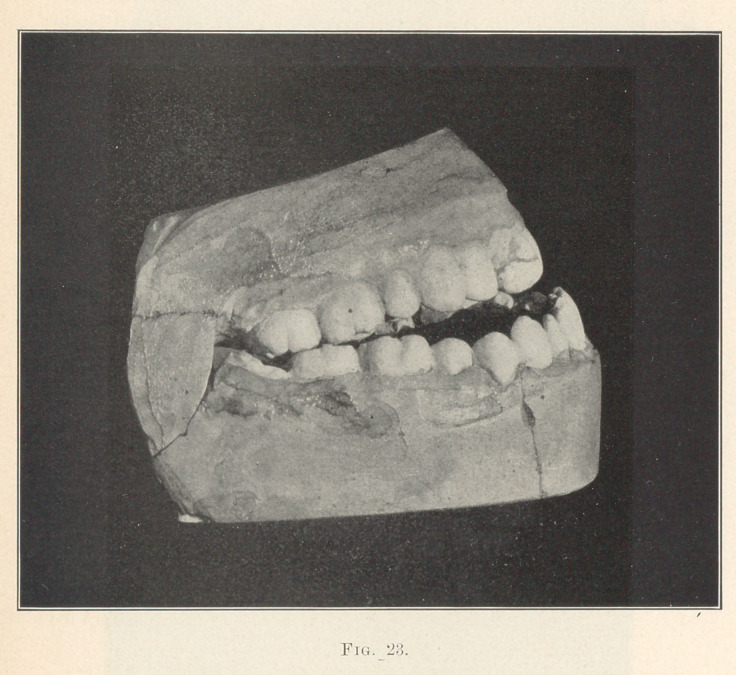


**Fig. 24. f24:**
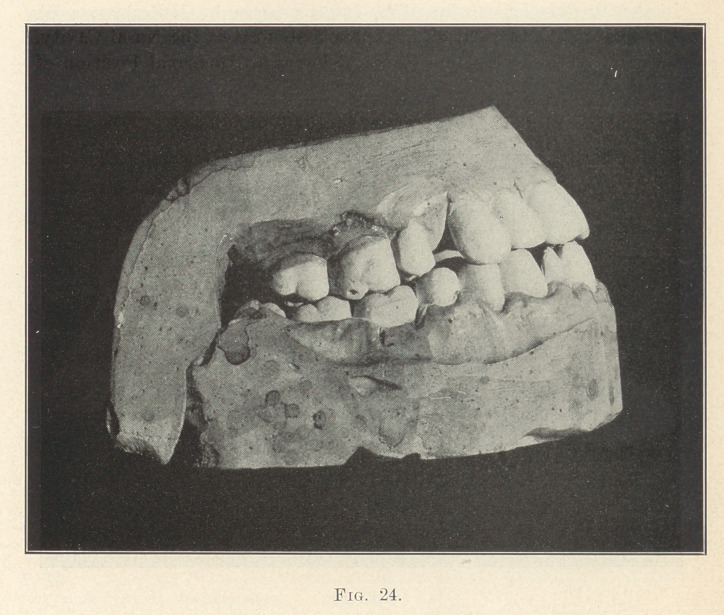


**Fig. 25. f25:**
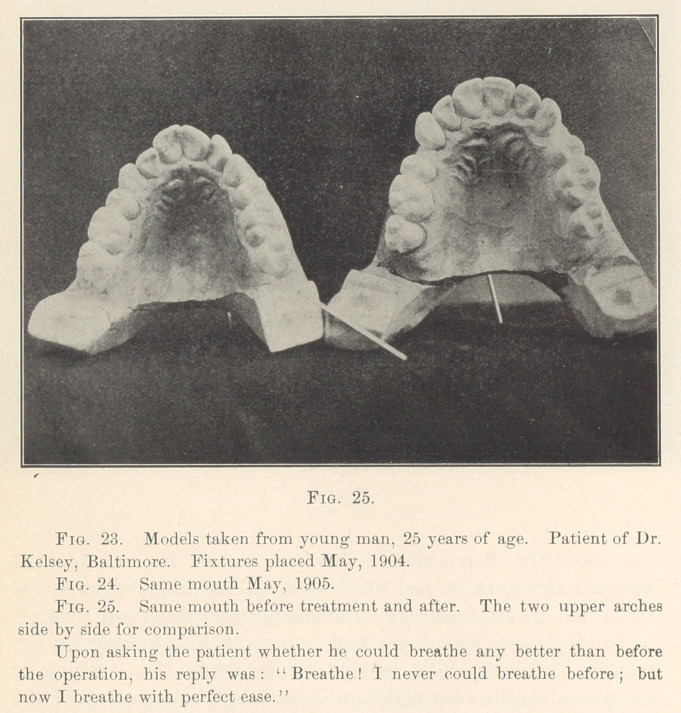


**Figs. 26 and 27. f26:**